# Patient complexity in quality comparisons for glycemic control: An observational study

**DOI:** 10.1186/1748-5908-4-2

**Published:** 2009-01-06

**Authors:** Monika M Safford, Michael Brimacombe, Quanwu Zhang, Mangala Rajan, Minge Xie, Wesley Thompson, John Kolassa, Miriam Maney, Leonard Pogach

**Affiliations:** 1 Deep South Center on Effectiveness at Birmingham VA Medical Center and University of Alabama at Birmingham, Birmingham, AL, USA; 2 VA New Jersey Healthcare System, East Orange, NJ, USA; 3 University of Medicine and Dentistry of New Jersey-New Jersey Medical School, Newark, NJ, USA; 4 Rutgers University, Piscataway, NJ, USA

## Abstract

**Background:**

Patient complexity is not incorporated into quality of care comparisons for glycemic control. We developed a method to adjust hemoglobin A1c levels for patient characteristics that reflect complexity, and examined the effect of using adjusted A1c values on quality comparisons.

**Methods:**

This cross-sectional observational study used 1999 national VA (US Department of Veterans Affairs) pharmacy, inpatient and outpatient utilization, and laboratory data on diabetic veterans. We adjusted individual A1c levels for available domains of complexity: age, social support (marital status), comorbid illnesses, and severity of disease (insulin use). We used adjusted A1c values to generate VA medical center level performance measures, and compared medical center ranks using adjusted versus unadjusted A1c levels across several thresholds of A1c (8.0%, 8.5%, 9.0%, and 9.5%).

**Results:**

The adjustment model had R^2 ^= 8.3% with stable parameter estimates on thirty random 50% resamples. Adjustment for patient complexity resulted in the greatest rank differences in the best and worst performing deciles, with similar patterns across all tested thresholds.

**Conclusion:**

Adjustment for complexity resulted in large differences in identified best and worst performers at all tested thresholds. Current performance measures of glycemic control may not be reliably identifying quality problems, and tying reimbursements to such measures may compromise the care of complex patients.

## Background

Patient complexity has recently been raised as an important issue in patient care and quality assessment [1-4]. While complexity from multiple medical conditions has been increasingly discussed [1-4], there are important additional sources of complexity that directly impact patient care. For example, patients' behavior and availability of psychosocial support mechanisms may directly impact clinical decision-making. The Vector Model of Complexity proposes that a patient's complexity arises out of interactions between six domains: biology/genetics, socioeconomics, culture, environment/ecology, behavior, and the medical system [5]. Currently, the only aspect of patient complexity included in quality assessments is patient age, because most performance measures for accountability exclude older individuals.

The influence of complexity on patient outcomes is well-demonstrated in diabetes, which has a number of accountability performance measures. Many diabetes patients have multiple medical problems, contributing to complexity along the Vector Model's biological vector. Additional challenges along this axis are imposed by disease severity, because diabetes can be easier to control early in its course. Complexity is also introduced along the behavioral axis, because diabetes imposes considerable self-care demands [6]. These self-care demands can be especially difficult for patients who lack social support, contributing complexity along the socioeconomic vector [7-9]. In the U.S. Department of Veterans Affairs (VA), married diabetic men have better glycemic control than unmarried diabetic men [10], demonstrating the importance of social support in men with diabetes. All of these sources of complexity alone or in combination call for clinical trade-off decisions, possibly deviating from 'ideal care.' When the Institute of Medicine recommended that the ideal health care system deliver care is 'driven by shared decision-making and based on continuous, healing relationships' [11], it acknowledged that for some patients, especially those that are complex, 'good care' will not necessarily lead to ideal performance measures.

None of these aspects of patient complexity is currently reflected in performance measures widely used for public reporting, and, more recently, to 'pay for performance' (P4P) [12-14]. While public reporting of the quality of healthcare has had measurable effects on improving population health [15-17], the fact that currently implemented performance measures do not account for patient complexity has raised concerns [1-4]. For diabetes, the public accountability measure has been an assessment of poor glycemic control as reflected by hemoglobin A1c >9.0%, with plans to set this threshold at lower levels [18]. This performance measure is based on well-established evidence that glycemic control is associated with diabetes outcomes [19]. However, it is unclear how patient complexity influences the assessment of quality of care provided by health care plans.

One issue that has prevented accounting for complexity in quality assessments is the lack of methods to do so. We studied how incorporating several readily available characteristics that reflect patient complexity along the biological vector (age, comorbidity, severity of diabetes as reflected in insulin use), and the socioeconomic vector (as reflected by marital status) affected assessments of quality of care. We conducted this study in the nation's largest integrated health system, the Veterans Health Administration (VHA).

## Methods

### Data

We used data from the VHA's Healthcare Data Analysis Information Group (Milwaukee, WI) for pharmacy data and A1c values, and the National Patient Clinical Dataset from the Veterans Integrated Service Network Support Center (Austin, TX) for inpatient and outpatient administrative utilization data with associated International Classifications of Diseases, 9^th ^edition (ICD-9) codes and demographic information. To identify diabetes, we selected veterans who used the VHA in 1998, were alive at the end of 1999, and in 1999 received either a diabetes medication or had an ICD-9 code 250.xx (diabetes) associated with more than one outpatient encounter or any inpatient encounter at one of the VHA's 145 medical centers [20].

When multiple values for A1c were present for an individual for 1999, we used the last available value. Values that fell above the physiologic range were excluded (>18.0%).

Several VA medical centers could not be included because of A1c lab assay methodology. Precision and bias problems with A1c laboratory assays to measure A1c levels have been reported [21-23], and a national effort to standardize A1c test methods is underway. Because standardization of A1c methodology was not mandated in the VHA until late in 1999, we contacted the laboratory director at each medical center to determine which A1c lab method was utilized at their medical center that year. Only the 66 medical centers using exclusively National Glycohemoglobin Standardization Project-certified methods were included in this study [24].

### Variables used to reflect patient complexity

According to the Vector Model of Complexity, patient complexity can arise along vectors represented by the major determinants of health: socioeconomic, cultural, biological, environmental, and behavioral [25]. Not all of these influences can be readily assessed using administrative data. Because our goal was to use existing data, we examined variables that were available in the VA databases. They included a variable along the socioeconomic vector (married status) and several variables along the biological vector, including age, comorbid illnesses and severity of diabetes approximated by insulin treatment. In type 2 diabetes, insulin treatment signals failure of oral therapies and reflects more advanced disease. Patients are often reticent to initiate treatment requiring needles and more intense monitoring, therefore insulin treatment in type 2 patients is an approximate indicator of disease severity [19]. To represent comorbid illnesses, we used Selim's Comorbidity Score, a validated method developed among veterans that sums the presence of any of 30 common chronic illnesses into a single unweighted score [26]. Selim's Comorbidity Score correlates with the physical component summary score of the SF-36 [27]. Both inpatient and outpatient utilization data and associated ICD-9 codes were used to construct the comorbidity score. We also tested the more widely used Deyo modification of the Charlson Comorbidity Index to represent comorbid conditions [28,29], and found similar results; therefore we present results only using Selim's Comorbidity Score.

### Modeling

We used linear regression models to complexity-adjust individual A1c levels as a continuous measure. Covariates included age, marital status, insulin treatment, and comorbidity score. Because an essential feature of the Vector Model is the interrelatedness of components of complexity, we considered interactions between age and the comorbidity score, and between age and diabetes treatment. We retained only variables that were significant at the *p *< 0.05 level in the final model.

We evaluated the model's performance by examining R^2 ^and by dividing the adjusted A1c values into deciles, then examining the proportion of the unadjusted above-threshold values in each decile [30]. We evaluated model stability by drawing 30 random 50% subsamples and examining their ranges of the regression coefficients and R^2^.

### Profiling

**Figure 1:**
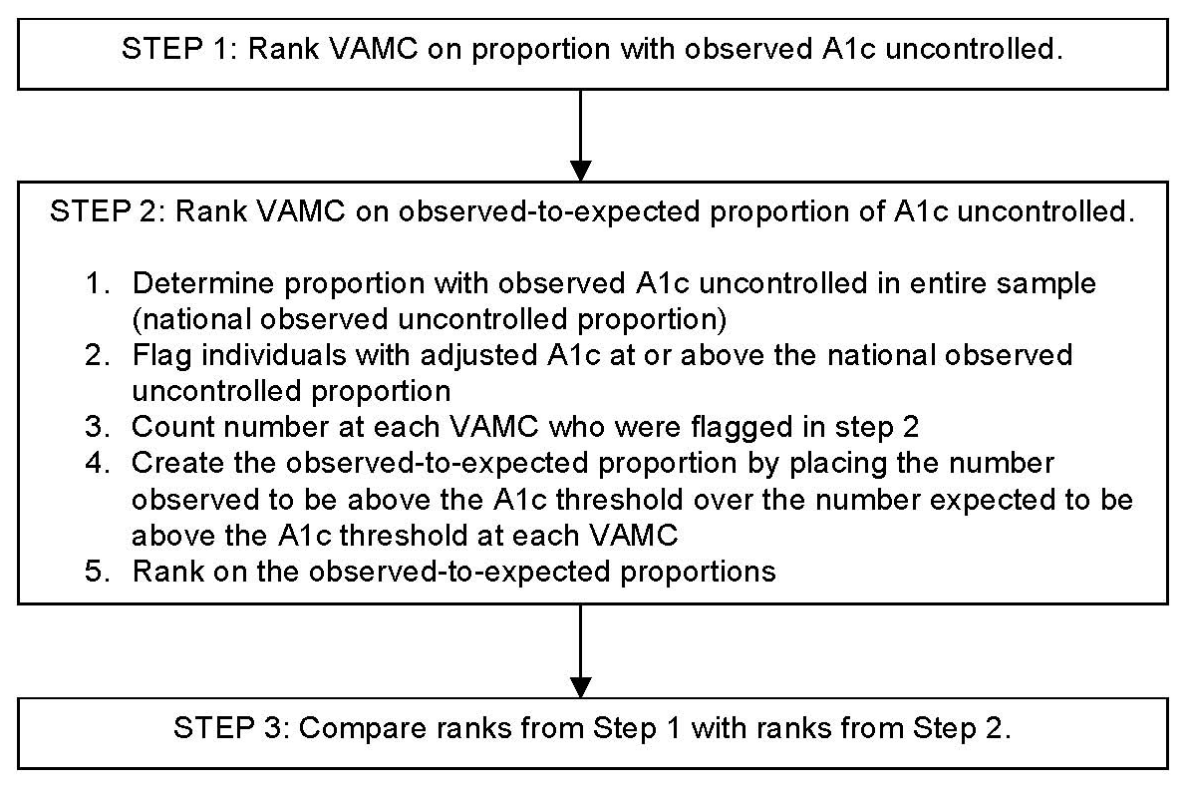
**Steps used to examine results of using unadjusted vs. adjusted A1c to rank VA Medical Centers on glycemic control**.

To evaluate the effect of complexity-adjustment on profiling (rank order), we proceeded in three steps (see Figure 1). In step one, we created 'observed,' or unadjusted ranks. We first created proportions for each VAMC consisting of the number of individuals with observed A1c at or above threshold divided by the total number of diabetes patients at that VAMC. We then ranked VAMC on these proportions.

In step two, we created 'observed-to-expected,' or adjusted, ranks. We first determined the entire national study population's (all 66 VA medical centers) proportion of observed uncontrolled patients (the national observed uncontrolled proportion). Then, we flagged individuals with adjusted A1c's at or above the corresponding national observed uncontrolled proportion for each threshold (the 'expected' uncontrolled). For example, 15.6% of patients at the 66 medical centers overall had unadjusted A1c >9.5%; we therefore identified all sample members with the 15.6% highest adjusted A1c values. Next, we counted the number of individuals at each VAMC who were flagged as being in the national observed uncontrolled proportion. We then used this number of 'expected' uncontrolled to create the 'observed-to-expected' ratio for each VAMC. Last, we ranked VAMC on these 'observed-to-expected' ratios.

In step three, we compared the results with ranks obtained in step two (adjusted ranks) with those obtained in step one (unadjusted ranks). We repeated this process for each of the tested thresholds of A1c: 8.0%, 8.5%, 9.0%, and 9.5%.

All statistical analyses were performed using STATA (Version 7.0, Stata Corporation, 2001) and SAS (Version 9.0, Cary, NC). The VA New Jersey Healthcare System institutional review board approved the study.

## Results

The patients in the study sample (n = 118,167) were similar to the overall VHA diabetes population (Table 1), with a mean age of 64 years (SD 11) and 63% married. Patients in the study sample had on average 1.7 more comorbid medical conditions than the population of veterans with diabetes. Thirty-nine percent were on insulin, either alone or in combination with oral agents. The 66 sample VA medical centers cared for a mean number of 1,790 patients with diabetes (range 328 to 5192) and 15.6% of the study sample had A1c >9.5% (range 4.9 to 25.2%), 21.7% >9.0% (6.7 to 32.1%), 29.6% >8.5% (11.9 to 42.3%), and 39.7% >8.0% (21.2 to 54.5%).

**Table 1:** Patient characteristics* of all VHA patients with diabetes and the study sample.

Patient characteristic	Veterans with diabetes	Study sample
N	552,128	118,167
Age in years, mean ± SD	64.2 ± 11.1	64.1 ± 11.1
Married, %	61.9	62.7
Diabetes treatment, %		
No VHA meds	3.3	3.2
Oral agents only	57.7	57.9
Insulin only	21.9	22.7
Insulin + oral agent(s)	17.2	16.2
Comorbidity score ± SD	3.4 ± 2.6	5.1 ± 3.0

*p-values not shown due to large sample and small differences reaching statistical significance. 98% of the sample was male.

Medical Center ranks differed modestly simply by using a different A1c thresholds, without any adjustment. Compared with ranks obtained using the 9.0% threshold, 51% of ranks obtained using 8.0% were within five ranks. For the best quartile of performance, 76% of ranks for the 8.0% threshold were within five ranks of those obtained using 9.0%.

### Modeling results

All variables and the age * comorbidity score interaction contributed significantly to variation in A1c and were retained in the final model (Table 2). This model's R^2 ^was 8.3%. The resampled coefficient means were very close to the original model with narrow ranges (Table 2), reflecting model stability.

**Table 2:** Complexity-adjustment model for A1c with model coefficients and coefficients of thirty random 50% subsamples (with resampling).

Variable	Model Coefficients ± SE	Mean of Resampled Coefficients [Range]
Age group (years)		
<55 vs. >75	0.92 ± 0.03	0.92 [0.84, 0.99]
55 – 65 vs. >75	0.49 ± 0.03	0.49 [0.39, 0.56]
65 – 75 vs. >75	0.21 ± 0.03	0.20 [0.14, 0.26]
Married vs. not	0.07 ± 0.01	0.07 [0.05, 0.10]
Insulin vs. no insulin	-0.75 ± 0.01	-0.75 [-0.78, -0.72]
Comorbidity score	-0.02 ± 0.00	-0.03 [-0.04, -0.02]
Age group * Comorbidity score		
<55 vs. >75	-0.05 ± 0.01	-0.05 [-0.07, -0.04]
55 – 65 vs. >75	-0.02 ± 0.01	-0.02 [-0.03, 0.00]
65 – 75 vs. >75	-0.01 ± 0.01	-0.01 [-0.02, -0.00]
Intercept	8.16 ± 0.02	8.16 [8.10, 8.23]
Model R^2^	0.083	0.084 [0.078, 0.089]

**Figure 2:**
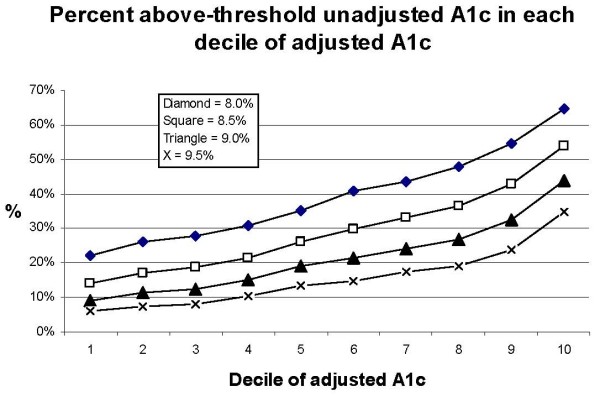
**Deciles of adjusted A1c with contribution of above-threshold unadjusted A1c**.

Decile of risk tables [30] indicated that each decile of adjusted A1c had successively more above-threshold observed values, as expected (Figure 2). Trends were similar for each threshold. The considerable change in the proportion of unadjusted above-threshold values across the deciles indicated that assessments based on unadjusted values were quite different from those based on adjusted values.

### Effect of complexity-adjusted A1c on VA medical center profiling

**Figure 3:**
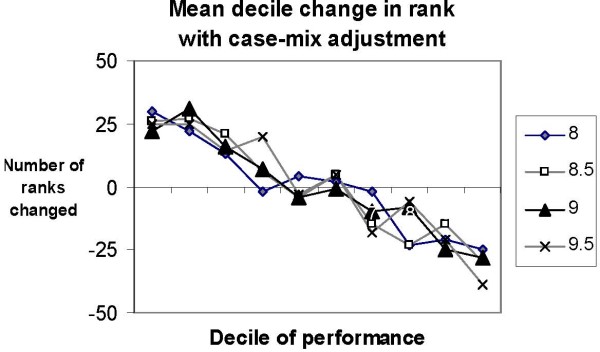
**Mean decile change in rank with adjustment for 66 VA medical centers**. Decile 1 includes the 'top' performers among these 66 VA medical centers. For each threshold of A1c, VA medical centers in Decile 1 experienced an average change in rank of 25.

The effects of adjusting for complexity on profiling were substantial. Figure 3 demonstrates that the greatest changes in rank occurred in the extreme deciles that are the focus of quality assessment. These changes were similar across all the thresholds tested. For medical centers in the best decile of performance, the average change in rank was 25, with similar magnitude of change for medical centers within the worst decile of performance. Table 3 depicts the actual rank changes experienced by the top and bottom ten performers. Between zero and two medical centers remained in the top ten after complexity-adjustment, and one to two medical centers remained in the bottom ten, depending on the threshold. Remarkably, two to three medical centers in the bottom ten became top ten performers with complexity-adjustment, regardless of the threshold chosen.

**Table 3:** Rank changes* with adjustment among the top ten and bottom ten performers among 66 VA medical centers

	Adjusted rank			
	
	8.0%	8.5%	9.0%	9.5%
Top ten performers, unadjusted rank				
**1**	30	**7**	**3**	29
**2**	11	33	31	20
**3**	40	40	30	11
**4**	56	34	48	**3**
**5**	36	56	**9**	56
**6**	29	17	35	38
**7**	39	23	28	46
**8**	51	42	50	33
**9**	22	41	32	37
**10**	37	48	43	28
Number among top ten performers, unadjusted, who would be ranked as top ten performers with adjustment	0	1	2	1

Bottom ten performers, unadjusted rank				
57	50	58	**5**	34
58	**4**	50	12	21
59	**3**	60	57	60
60	58	51	59	**10**
61	52	53	53	**5**
62	57	**3**	56	55
63	**8**	55	**4**	**2**
64	20	16	23	26
65	38	**6**	44	27
66	54	49	**6**	44
Number among bottom ten performers, unadjusted, who would be ranked as *top *ten performers with adjustment	2	2	3	3

*Emboldened numbers indicate adjusted ranks in the top ten.

## Discussion

Using A1c values adjusted for only a few domains of patient complexity as proposed in the Vector Model of Complexity caused substantial differences in which VA medical centers were identified as best and worst performers. Depending on the threshold, 20–30% of the ten best performers among these 66 medical centers using adjusted values would have been identified as among the ten worst performers without adjustment. Our findings suggest that patient complexity has a major impact on quality assessment using unadjusted A1c in the glycemic control performance measure. This finding calls into questions whether currently used methodology should be tied to reimbursement [13]; one of the indicators currently included in the Bridges to Excellence diabetes program for physicians and providers includes the proportion of people with diabetes who had poorly controlled A1c levels at last measure.

Adjustment for complexity had a similar impact across all tested thresholds of A1c. Although our study focuses on thresholds in place at the time of the study (A1c >9.0%), our findings have implications for the recent decision to lower the threshold for glycemic control by National Committee on Quality Assurance, since the plans are to use unadjusted A1c levels [18].

We were unable to include important aspects of patient complexity, along the behavioral, cultural, and environmental vectors. We only had one element along the socioeconomic vector, and even along the biological vector, the elements were not comprehensive. For example, we examined the role of specific illnesses, including mental health conditions, on quality of care assessments, and found that the relationship between achieving optimal glycemic control and specific comorbid illness patterns is heterogeneous [31]. In addition, ICD-9 codes cannot reflect the severity of conditions. Some quality assessments currently rely on patient surveys, and we have shown that survey-derived data captures important additional dimensions with profound impact on quality of care assessments [32]. Surveys are not without their drawbacks, including biases and cost [33]. It is clear that thoughtful approaches to capturing the full picture of patient complexity are needed.

Nevertheless, our study's findings of large differences in identified best and worst performing medical centers underscores the urgency in incorporating complexity, even in rudimentary form, into quality comparisons. Because of the inevitable trade-off decisions required in the care of complex patients, their care may not be assessed as 'guideline concordant.' Tying guideline concordant care to reimbursement creates a tension for treating physicians: should they 'treat for performance' or treat the patient? Both may not be possible; P4P could potentially pose a threat to the overall quality of care received by complex patients, as perverse incentives encourage the clinician to spend less time engaging the patient in eliciting preferences and developing congruence on a tailored treatment plan.

The rudimentary variables we did include in our adjustment model are widely available, which could be abstracted from medical records during annual data collection for performance measures. The model demonstrated explanatory value in a range typical for so-called case-mix adjustment models [30], the deciles of adjusted values had the expected increasing contribution of above-threshold unadjusted values, and the parameter estimates were stable with repeated sampling. Further, the simplicity of our approach makes it feasible in various settings.

Our study was conducted in the VA, which includes largely older men who may be more debilitated than the general population. Our findings should be examined in other populations. In addition, while the components of complexity changed assessed performance, our study was not designed to assign 'appropriateness' to elements of complexity for quality of comparisons, an area of ongoing clinical debate.

## Conclusion

Adjusting A1c levels for readily available characteristics that reflect some aspects of patient complexity resulted in large differences in identifying best or worst performers, most pronounced at the extremes of performance that are the focus of quality assessment. These findings were similar across all tested thresholds of A1c, suggesting that both domains of patient complexity included in this study were important influences at all levels of glycemic control. It is not clear to what extent current practices for assessing glycemic control as a quality of care indicator may be identifying differences in the populations the health systems serve, or differences in the quality of care they provide. Tying such measures to reimbursement may not be in the best interest of patients until a measure more convincingly reflective of quality of care can be proposed.

## Competing interests

The authors declare that they have no competing interests.

## Authors' contributions

MS conceived of the paper, interpreted data, drafted the manuscript and procured funding. MB provided conception and design input, conducted the analyses and participated in providing critical revisions to the draft. QZ provided conception and design input, as well as critical revisions to the draft. MR provided conception and design input, and acquired data. MX, WT, and JK provided conception and design input, and provided critical revisions to the draft. MM conducted analyses and provided critical revisions to the draft. LP provided input into conception of the paper, interpreted data, critical revisions to the manuscript and procured funding.
